# Characterizing eating behavioral phenotypes in mood disorders: a narrative review

**DOI:** 10.1017/S0033291722002446

**Published:** 2022-10

**Authors:** Elena Koning, Jacob Vorstman, Roger S. McIntyre, Elisa Brietzke

**Affiliations:** 1 Centre for Neuroscience Studies (CNS), Queen's University, Kingston, ON, Canada; 2 Program in Genetics and Genome Biology, Research Institute, The Hospital for Sick Children, Toronto, ON, Canada; 3 Department of Psychiatry, University of Toronto, Toronto, ON, Canada; 4 Mood Disorders Psychopharmacology Unit (MDPU), Toronto Western Hospital, University Health Network, Toronto, ON, Canada; 5 Department of Psychiatry, Queen's University School of Medicine, Kingston, ON, Canada

**Keywords:** Bipolar disorder, eating behavior, major depressive disorder, mood disorders, nutrition, phenotype

## Abstract

Mood disorders, including depressive and bipolar disorders, represent a multidimensional and prevalent group of psychiatric illnesses characterized by disturbances in emotion, cognition and metabolism. Maladaptive eating behaviors in mood disorders are diverse and warrant characterization in order to increase the precision of diagnostic criteria, identify subtypes and improve treatment strategies. The current narrative review synthesizes evidence for Eating Behavioral Phenotypes (EBP) in mood disorders as well as advancements in pathophysiological conceptual frameworks relevant to each phenotype. Phenotypes include maladaptive eating behaviors related to appetite, emotion, reward, impulsivity, diet style and circadian rhythm disruption. Potential treatment strategies for each phenotype are also discussed, including psychotherapeutic, pharmacological and nutritional interventions. Maladaptive eating behaviors related to mood disorders are relevant from both clinical and research perspectives, yet have been somewhat overlooked thus far. A better understanding of this aspect of mood disorders holds promise to improve clinical care in this patient group and contribute to the subtyping of these currently subjectively diagnosed and treated disorders.

## Introduction

Mood disorders, including major depressive disorder (MDD) and bipolar disorders (BD), are leading causes of disability worldwide (McIntyre et al., 2020; Steel et al., 2014). Their burden is not limited to mood and emotional disturbances but extends to multiple domains including cognition, sleep and appetite (Malhi & Kuiper, 2013; McIntyre et al., 2013; Rakofsky & Rapaport, 2018). One intriguing aspect of the presentation of mood disorders is related to changes in metabolism and eating (Barandas, Landgraf, McCarthy, & Welsh, 2015). Different maladaptive eating behaviors are already being observed in MDD and BD and have even been incorporated into the DSM-5 diagnostic criteria for depressive episodes. However, none of the diagnostic criteria addresses eating behavior regarding manic or hypomanic episodes of mood disorders (Ringeisen et al., 2016). Metabolic syndrome, obesity and type II diabetes are more common in mood disorders when compared to the general population (SayuriYamagata, Brietzke, Rosenblat, Kakar, & McIntyre, 2017). Increased appetite does not fully account for the heterogeneity in findings. For example, some individuals with depression exhibit undereating and sub-optimal weight status while others demonstrate high appetite and adiposity. The mechanism underlying patterns of appetite change remains largely unknown (Simmons et al., 2020).

Eating Behavioral Phenotypes (EBP) can be defined as a set of eating behaviors that are characteristic of an individual or group (Bouhlal, McBride, Trivedi, Agurs-Collins, & Persky, 2017). Convergent findings from multiple lines of evidence suggest that a bi-directional relationship exists between eating behaviors and mood, manifesting as overlap between maladaptive mood and eating behavior. Despite this, the characteristics of the observed changes in eating patterns in individuals with MDD and BD remain poorly investigated. An improved understanding of the interaction between eating patterns and core mood symptoms may provide an opportunity for new interventions to be developed.

It has been suggested that distinguishing different eating behaviors in mood disorders will improve both diagnostic accuracy and treatment efficacy through subtyping and precision medicine approaches (Mills, Thomas, Larkin, Pai, & Deng, 2018; Paans et al., 2018; Simmons et al., 2016). However, there is yet to be a review article comprehensively discussing the implications of different EBPs for clinical care and research. Therefore, the purpose of the current narrative review is to present evidence for and characterizations of EBPs in mood disorders as well as discuss potential interventional approaches based on each phenotype.

## The concept of eating behavioral phenotypes

In healthy individuals, eating behaviors are adaptive and important for homeostasis. For example, it is well established that when the body is in a constant state of negative or positive energy balance, homeostatic mechanisms function to alter appetite and restore fat levels to normal (Casanova, Beaulieu, Finlayson, & Hopkins, 2019). This negative feedback mechanism explains why dieters who have recently lost weight tend to eat larger meals and more daily calories, causing weight gain until regaining their so-called ‘set point’ again (Woods & Ramsay, 2011). These homeostatic controls are thought to incite adaptive behavioral responses including motivation, locomotion, cognition, and mood changes (Ferrario et al., 2016; Wynne, Stanley, McGowan, & Bloom, 2005). Under pathological conditions, eating behaviors can become more stereotyped and less adaptive, being part of the confluence of behavioral manifestations seen in mood disorders (Goldschmidt et al., 2014; McAulay, Hay, Mond, & Touyz, 2019).

Phenotyping is a biological concept in which patterns are identified between observable traits and physiological mechanisms, such as the factors which determine eating behavior in humans (Bryan et al., 2017). Phenotyping was first used in genetic research, but is also important to clinical medicine as a tool for treatment stratification. In essence, such approach aims to select a subset of individuals who are most likely to benefit from a specific treatment modality, based on the identification of a common physiological underpinning for their observable symptoms (Lussier & Liu, 2007; Robinson, 2012). However, only a handful of studies have applied EBPs to groups of individuals with medical conditions (Bouhlal et al., 2017). For example, individuals with obesity have been classified based on their eating behaviors in which emotional eating is more associated with neurocognitive impairment and affective disorders when compared to other eating behaviors (Caroleo et al., 2018). Some individuals with chronic pain report increased emotional eating or binge eating in response to pain while others report reduced appetite and weight loss (Amy Janke & Kozak, 2012; Pilgrim, Robinson, Sayer, & Roberts, 2015). Different eating phenotypes are also observed in thyroid disease (Amin, Dhillo, & Murphy, 2011; Gonzalez-Torres & Dos Santos, 2019; Yau & Potenza, 2013). The biological basis of EBPs is complex and regulated at multiple levels, including appetite and satiety control, the reward system, the sleep-wake circadian rhythm and the nutritional content of the diet, as outlined in Table 1.

**Table 1.** Key aspects of human physiology involved in the regulation of eating behavior

Eating behavior regulator	Relevant mechanisms
Appetite and satiety	Neuroendocrine signaling Neuroendocrine signals → appetite-stimulating ARC neurons → Hunger Neuroendocrine signals → appetite-suppressing ARC neurons → Satiety Pancreas → Insulin → Hypothalamus → Inhibition of food Intake Adipose tissue → Leptin → Hypothalamus → Inhibition of food Intake
	Psychological factors Negative body image/self-esteem → Increased appetite Negative affect → Increased or decreased appetite Cognition → Increased or decreased appetite
	Social factors Early life experiences and culture influences Socio-economic status Social context
	Genetic factors Leptin deficiency → Increased appetite + hyperphagia MC4R mutation → Abnormal leptin activity → Uncontrolled eating Polymorphisms of taste receptors → Appetite for different tastes Hypothalamic development mutation → Disinhibition emotional eating
The reward system	Increased leptin → Reduced dopaminergic neuron signaling → Satiety Reduced leptin → Increased dopaminergic neuron signaling → Hunger maladaptive eating behavior → Dysfunction reward system activity → Food addiction
The circadian system	Feeding → Peripheral clocks → Signals → Hypothalamus → Physiology → Behavior Irregular meal timing → Disturbed metabolic gene and neuroendocrine oscillation → Uncoupling from the light cycle → Physiology → Maladaptive behavior
Nutritional content	High-carb diet → Increased appetite Highly palatable foods → Increased reward system activity → Increased Appetite High-protein diet → Reduced Appetite High-fat diet → Dysfunctional circadian and reward system activity and altered gut microbiome → Reduced satiety → Weight gain Western diet → Gut microbiome dysbiosis → Dysfunctional Physiology → Behavior Plant-based/Mediterranean diets → Diverse gut microbiome → Beneficial Physiology → Behavior

ARC, Arcuate Nucleus; MC4R, Melanocortin 4 Receptor.

Appetite is a complex sensation involving the integration of multiple peripheral signals and brain regions to maintain an optimal energy supply. The main controller of appetite in the brain is the arcuate nucleus (ARC) of the hypothalamus which projects to regions involved in emotion, reward and memory (Subramaniapillai & McIntyre, 2017; Wynne et al., 2005). Various neuroendocrine signals are released from the digestive tract and act on appetite-suppressing or appetite-stimulating neurons of the ARC to regulate appetite. Conversely, the long-term regulation of appetite occurs in response to adiposity levels, through signaling molecules such as insulin and leptin (Wynne et al., 2005).

Hedonic aspects of eating also significantly control behavior and there is significant overlap in neural pathways responsible for appetite and reward, including dopaminergic neurons of the ventral tegmental area (Alonso-Alonso et al., 2015). This system serves the evolutionary role of motivating an organism to eat during energetic deficits and to pursue other rewards when fat stores are high. Reward circuits respond to information from taste buds and eating-related neuroendocrine signals to control an organism's motivation to feed (Figlewicz & Sipols, 2010; Leng et al., 2017; Mansur et al., 2019). Signals such as leptin and insulin play a significant role in the effects of metabolic status on reward pathways in addition to opioids, endocannabinoids and serotonin (Figlewicz & Sipols, 2010; Wynne et al., 2005).

Another relevant regulator of eating behavior is the circadian clock system. This system optimizes the timing in which energetic processes occur to fulfill an organism's energy needs which oscillate across the day-night cycle (Laermans & Depoortere, 2016; Pickel & Sung, 2020). The timing of food intake is arguably the strongest entrainment cue for the periphery. Digestive tissues even demonstrate circadian oscillations in digestive and metabolic capacity (Aoyama & Shibata, 2020; Laermans & Depoortere, 2016). Circadian oscillations are also observed in gut microbiome composition, hormone function and reward system signaling (Blancas-Velazquez, Mendoza, Garcia, & la Fleur, 2017). Irregular meal timing is associated with disturbed oscillations of metabolic genes, altered appetite-regulating hormones and maladaptive feeding behaviors (Li et al., 2020; Maury, 2019; Reid, Baron, & Zee, 2014). In a bi-directional manner, food intake patterns also influence the circadian system. For example, diet-induced obesity increases eating in the resting phase, causing disturbed clock gene function and neuroendocrine rhythms (Blancas-Velazquez et al., 2017; Guerrero-Vargas, Espitia-Bautista, Buijs, & Escobar, 2018; McHill et al., 2017).

Finally, eating behavior is largely subjected to the effects of nutritional content. For example, individuals who consume a lunch high in carbohydrates exhibit altered eating behavior at dinner when compared to a high-protein meal (Latner & Schwartz, 1999). In addition, protein intake results in reduced hunger while diets low in protein induce cravings for savory foods (Carreiro et al., 2016). Conversely, a high-fat diet (HFD) has been shown to distort the circadian rhythms of neuroendocrine signals as well as alter clock gene function (Carreiro et al., 2016; Kohsaka et al., 2007; Maury, 2019).

**Fig. 1.**
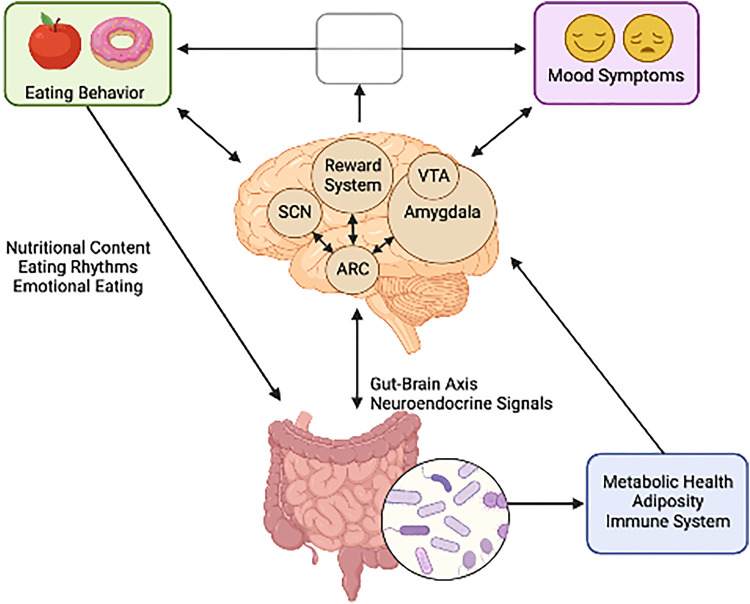
The bi-directional relationship between neural circuits governing mood and feeding behavior. (Figure created with BioRender.com).

All the processes involved in the regulation of eating behavior are elements of an orchestra in which the brain is the conductor, integrating these factors and generating different behavioral phenotypes as a result. Consequently, one could expect that mood-related alterations in brain function change the music, i.e., the conductor may influence and be influenced by patterns of food intake. As an essential behavior to human survival, many systems have evolved to regulate feeding, including emotional processes, as depicted in Fig. 1. There is anatomical overlap between neural circuits regulating feeding and emotion, including neurons of the hypothalamus, amygdala and ventral tegmental area (Sweeney & Yang, 2017). Conversely, eating behavior has also been shown to impact mood through the immune system and gut brain-axis (Firth, Gangwisch, Borisini, Wootton, & Mayer, 2020). These mechanisms may also explain the common comorbidity between eating disorders (EDs) and mood disorders which is well supported in the literature (DeSocio, 2019; Godart et al., 2015; McAulay et al., 2019). A greater understanding of the directionality of the food-mood relationship is needed. The characterization of EBPs in mood disorders will contribute by identifying the behavioral correlates in which mood and eating are related.

## Eating behavioral phenotypes in mood disorders

**Fig. 2.**
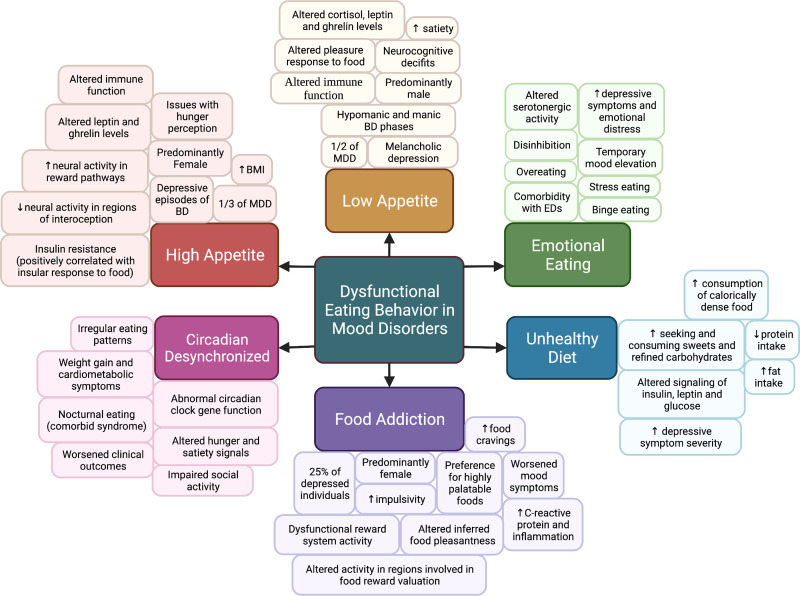
Various maladaptive eating behaviors and characteristics of Eating Behavioral Phenotypes (EBPs) in mood disorders. (Figure created with BioRender.com).

Different EBPs in mood disorders have been described but their biological underpinnings remain to be fully uncovered. In this section, we develop a preliminary characterization of some specific EBPs in mood disorders, as illustrated in Fig. 2. These EBPs were reached by reviewing the literature on eating behaviors in the mood disorder population and characterizing each behavior into distinct groups. Associations between groups of behaviors and certain characteristics of individuals with mood disorders were then identified, based on current evidence from the literature to date, including preliminary studies, systematic reviews and clinical trials. The existence of these EBPs will potentially support future investigations on the empirical characterization of eating behavior in mood disorders.

### The high appetite EBP

Approximately one third of individuals with depression exhibit increased appetite (Simmons et al., 2020). In individuals with BD, appetite may demonstrate a biphasic nature, often mirroring changing mood symptoms. For example, hypomania and mania have been associated with anorexia, hypophagia, and weight loss, while atypical depression has been associated with hyperphagia and weight gain (McElroy, Kotwal, Keck, & Akiskal, 2005).

The high appetite phenotype in mood disorders is characterized by the following aspects:

1. Predominance in females (Mills et al., 2018).
2. Deficits in interoception, described as the awareness of visceral cues such as hunger. It is associated with activity in the insula, a brain region that exhibits altered neural activity in individuals with depression-related appetite changes (Cosgrove et al., 2020). Individuals with this EBP demonstrate reduced neural activity in the insula (Simmons et al., 2016).
3. Difficulties in hunger perception and reward: Individuals with BD demonstrate greater perceived hunger and a difficulty eating healthily when compared to controls, especially BD type I (Seixas et al., 2012). Altered activity in the reward system in individuals with high appetite may indicate a basis for increased hunger, in which more food may be needed to elicit an optimal pleasure response to eating (Cerit et al., 2019).
4. Sex-specific abnormal hormonal responses: Females with increased appetite and weight gain demonstrate higher leptin levels when compared to those with reduced appetite while males demonstrate the opposite result (Mills et al., 2018). Depressed individuals with increased appetite also have increased ghrelin levels in response to a meal and this has been associated with increased activity in the reward system (Cerit et al., 2019). Additionally, insulin resistance is characteristic of this EBP in which the magnitude of resistance is positively correlated with neural responses of the insula to food cues (Simmons et al., 2020).
5. Altered immune function: Altered immune factors, including elevated levels of pro-inflammatory cytokines, are observed in individuals with depression (Goyal, Srivastava, Kodange, & Bhat, 2017; Leonard, 2010). Evidence suggests that cytokines influence appetite via the interoceptive pathways to the insular cortex (Andreasson, Arborelius, Erlanson-Albertsson, & Lekander, 2007).

### The low appetite EBP

Approximately half of individuals with depression exhibit reduced appetite and pathological weight loss as a result (Simmons et al., 2020). The low appetite EBP is characteristic of melancholic depression, a subtype characterized by anhedonia, psychomotor disturbance and low mood reactivity (Spanemberg et al., 2014). Importantly, appetite and weight restoration are some of the most reliable signs of remission in this patient group (Kazes, Danion, Grange, Pradignac, & Schlienger, 1993). This EBP is also observed in the hypomanic and manic phases of BD (McElroy et al., 2005).

The low appetite phenotype in mood disorders is characterized by the following aspects:

1. Predominance in males (Mills et al., 2018).
2. Altered perception of satiety and reward: Individuals with melancholia-related appetite decreases demonstrate increased satiety and reduced hunger when compared with healthy controls. In addition, individuals with melancholic depression have an altered pleasure response to food, which may parallel mechanisms of anhedonia (Andreasson et al., 2007; Kazes et al., 1993).
3. Abnormal hormone responses: Individuals with melancholic depression and decreased appetite have higher cortisol levels which are correlated with neural responses to food cues. This has been described as a main differentiating factor among depression subtypes (Simmons et al., 2020). A recent study in individuals experiencing a manic episode of BD found decreased levels of the appetite-stimulating hormone ghrelin when compared to healthy controls (Abdel Aziz et al., 2022).
4. Altered immune function: Elevated cytokine levels in mood disorders may cause orexigenic appetite regulatory mechanisms to be inhibited. Additionally, increased TNF-*α* levels are linked to increased depressive symptoms and low appetite in depression (Andreasson et al., 2007).
5. Neurocognitive deficits: Reduced appetite is especially common in late-life depression and is directly associated with neurocognitive performance. Low vitamin D or iron as a result of reduced food intake is implicated in this connection as well as overlapping neural circuits involved in mood and neurocognition (Potter, McQuoid, & Steffens, 2015).

### The emotional eating EBP

Emotional eating has been identified as the mediating factor between depression and weight gain, being a predictor of increased body mass index (BMI) and waist circumference in individuals with MDD (Goldschmidt et al., 2008; Konttinen, van Strien, Mannisto, Jousilahti, & Haukkala, 2019; van Strien, Konttinen, Ouwens, van de Laar, & Winkens, 2020).

The emotional eating phenotype in mood disorders is characterized by the following aspects:

1. Overeating: Individuals with mood disorders have an increased tendency to overeat in response to negative emotions (Frayn, Livshits, & Knauper, 2018).
2. A correlation exists between the severity of emotional eating and the severity of mood symptoms (Goldschmidt et al., 2014; Konttinen et al., 2019; Mills et al., 2018).
3. Comorbidity with EDs, especially binge eating disorder: As a shared characteristic between mood disorders and EDs, emotional eating is a potential mediator for the association between these two groups of conditions. It is estimated that about 20% of adults with mood disorders meet the criteria for binge eating disorder, more commonly in BD than in MDD (Woldeyohannes et al., 2016). Binge eating is strongly linked to increased BMI and a greater risk of suicidality, mood instability and substance use. It is well accepted that binge eating and obesity are prevalent in BD and are associated with an increased disease burden (Martin et al., 2016; McElroy et al., 2013). Individuals with BD and comorbid EDs may also exhibit self-injuring and impulsive behaviors and are more likely to be diagnosed with personality disorders and substance use (Balzafiore et al., 2017). The negative mood states present in mood disorders have been shown to predict a binge, whereas positive mood states reduce the likelihood of a bingeing episode. It is thought that binge eating provides a brief elevation in mood, suggesting that patients eat as a form or temporary self-regulation of affective state (Christensen, 1993; Polivy & Herman, 2005). Although research indicates significant overlap between binge eating and mood disorders, overlap with other EDs such as anorexia nervosa and bulimia nervosa has also been suggested in the literature (Godart, Perdereau, Jeammet, & Flament, 2005; Godart et al., 2007; McAulay et al., 2019).
4. Disinhibition: A loss of control over eating has been related to the increased prevalence of binge eating in this patient group (Bernstein, Nierenberg, Deckersbach, & Sylvia, 2015).
5. Altered serotonergic signaling: Reduced serotonin activity is related to both increased emotional eating and depressive symptoms. Specifically, decreased serotonin activity was observed in adolescents of the 5-HTTLPR genotype and linked to the aforementioned symptoms (Paans et al., 2018).

### The food ‘addiction’ EBP

Food addiction as a psychiatric diagnosis is heavily debated in the literature, however, it is evident that a subset of individuals with mood disorders exhibit addiction-like behaviors towards food. Specifically, a study indicated that approximately 25% of depressed individuals met the criteria for food addiction, and this occurred exclusively in females (Mills et al., 2018). Preliminary data suggest an inverse relationship between obesity and substance abuse, suggesting the presence of common underlying substrates (McIntyre et al., 2007).

The food ‘addiction’ EBP in mood disorders is characterized by the following aspects:

1. Increased impulsivity: The excessive engagement in pleasurable yet risky activities is characteristic of the food ‘addiction’ EBP (Martin et al., 2016). Similar levels of impulsivity have been observed in EDs, mood disorders, and substance use disorders (Bushnell, Wells, & Oakley-Browne, 1996).
2. Presence of food cravings: Individuals with BD report more frequent food cravings, mostly for foods high in fat, sodium and refined carbohydrates. In addition, the presence of more frequent food cravings in BD is associated with increased depressive symptoms and negatively correlates with ghrelin levels (Platzer et al., 2020).
3. Excessive consumption of highly palatable foods: Palatable foods are known to activate the brain's reward circuitry similarly to drugs of abuse and dopamine signaling impacts both food intake and mood (Singh, 2014).
4. Dysfunctional activity in the brain's reward system: Dysfunctional reward system activity is strongly associated with addiction. Regions involved in food significance and reward valuation, including the striatum and orbitofrontal cortex exhibit altered activity in depressed individuals with increased appetite (Cosgrove et al., 2020). In addition, increased appetite in MDD has been linked to increased C-reactive protein levels in the blood as well as functional coupling between the orbitofrontal cortex and insula. Inferred food pleasantness was also related to these changes in neural activity and inflammation, potentially related to food ‘addiction’ in mood disorders (Cosgrove et al., 2020).

### The unhealthy diet EBP

The nutritional content of a meal is known to influence mood, therefore, diet-related EBPs indicate a subset of individuals with mood disorders and broadens the potential for interventions based on improving symptomology through diet content (Marx, Moseley, Berk, & Jacka, 2017). Individuals with mood disorders routinely exhibit differential consumption of proteins, fats, and carbohydrates when compared to the general population (Godos et al., 2020; Oh, Yun, Chae, & Kim, 2020).

The unhealthy diet EBP is characterized by the following aspects:

1. Low protein intake: Reduced protein consumption is associated with a significantly increased risk of depression when compared to a normal intake of protein. In addition, when calories from protein are increased by 10%, the prevalence of depression is significantly reduced (Oh et al., 2020).
2. High intake of fats: Fat intake is linked to worsened mood disorder outcomes. Chronic high-fat feeding has been shown to promote negative emotional states including anxiety and depression in both animal and human studies.
3. High intake of calorically dense foods: A positive correlation exists between depressive symptom severity and an increased intake of energy-dense foods (Mills et al., 2018; Polivy & Herman, 2005).
4. Preference for sweets and refined carbohydrates: Studies have indicated that an increased consumption of processed foods and a lower consumption of essential nutrients is associated with depressive symptoms. In addition, sucrose consumption is higher in mood disorders when compared to the general population (Martin et al., 2016). Individuals with BD report a significantly higher consumption of sucrose, sweetened drinks and greater overall calorie intake when compared to the general population (Lopresti & Jacka, 2015). Carbohydrate craving has also been associated with fatigue, depression, paranoia, and disordered eating behaviors and is commonly observed in seasonal affective disorder (SAD), obesity and unipolar depression (Christensen & Pettijohn, 2001; Yang et al., 2020). Some evidence indicates that carbohydrate consumption leads to short-term mood enhancement, but increases psychological distress in the long-term. For example, one study described reduced cortisol levels immediately following the consumption of sweets in individuals with BD, an effect that did not last (Lopresti & Jacka, 2015). Eliminating sucrose from the diets of emotionally distressed individuals was associated with reduced distress and depressive symptoms in a different study (Christensen & Pettijohn, 2001). Despite these findings, more research is needed to confirm a mechanistic role for carbohydrate consumption on mental well-being.

### The circadian desynchronized EBP

Feeding is an important peripheral cue for the circadian system. Desynchronization between the circadian clock and feeding times is strongly associated with negative health outcomes, including depressive and anxiety symptoms in animal models of shift work (Guerrero-Vargas, Zarate-Mozo, Guzman-Ruiz, Cardenas-Rivera, & Escobar, 2021). In humans, desynchronized eating rhythms result in abnormal metabolic hormone signaling and weight gain (Blancas-Velazquez et al., 2017; Brum, Filho, Schnorr, Bottega, & Rodrigues, 2015; Dollet & Zierath, 2019). Eating rhythm disruption is highly related to worsened BD symptoms and is a causal factor in the high prevalence of cardiovascular diseases in this patient group, even when a normal number of calories are consumed (Asterholm & Scherer, 2012; Barandas et al., 2015; Gonzalez, 2014; Li et al., 2020). As a system that is heavily involved in the regulation of energy, misalignment between the circadian clock and meal timing promotes the suboptimal utilization of energy (Oike, Sakurai, Ippoushi, & Kobori, 2015).

The circadian desynchronized EBP in mood disorders is characterized by the following aspects:

1. Irregular eating patterns: Skipping meals or delaying breakfast is associated with depression while only eating one meal a day is associated with BD (Lopresti & Jacka, 2015; Vadnie & McClung, 2017; Wilson et al., 2020; Zhu et al., 2019). A positive relationship exists between the irregularity of eating and severity of hypomanic symptoms, including a strong correlation between irregular eating and length of manic symptoms (Buyukkurt et al., 2021).
2. Nocturnal eating: It is known that evening preference is common in individuals with BD and is a risk factor for the development of depression (Chung et al., 2012; Kitamura et al., 2010). Circadian misalignment as an EBP in mood disorders may also manifest as nocturnal eating syndrome, characterized by reduced feeding during the day and increased feeding at night. Depressed mood is also characteristic of nocturnal eating syndrome, especially in the evening and this disorder often involves nocturnal awakening with conscious episodes of compulsive food intake (Melo et al., 2018). Nocturnal eating syndrome is more prevalent in BD than in the general population and individuals with both diagnoses demonstrate more anxiety and worsened sleep behavior (Melo et al., 2018).
3. Altered social activity: Eating alone is strongly associated with irregular meal timing and mood disorders. It has been proposed that the effects of altered mood states on social activity may impair the timing of food intake (Lopresti & Jacka, 2015).
4. Neuroendocrine signaling: It has been suggested that altered hunger and satiety signals may cause disordered meal timing as these signals interact with the circadian system (Buyukkurt et al., 2021).

## Interventions based on eating behavioral phenotypes in mood disorders

With the identification of various EBPs in mood disorders and their direct relation to negative clinical outcomes, a first step is to refine the assessment of eating behaviors. Although questionnaires for tracking eating behavior are already available, these methods can often be confounded by recall bias and the limitations of human memory (Wisniewski, Henson, & Torous, 2019). Conversely, ecological momentary assessment methods allow for more reliable tracking of eating behaviors in real-time, while also reducing participant burden. Dietary content can be assessed with these methods through active data collection with a smartphone application or online website (Levinson et al., 2018; McIntyre et al., 2021; Wenze & Miller, 2010). Furthermore, smartphone apps and wearable trackers are beginning to be implemented in psychiatric research in order to gain a better understanding of the clinical and pathophysiological aspects of mood disorders (Jagesar, Vorstman, & Kas, 2021; Jongs et al., 2020).

A second step is the promotion of a healthy diet. Dieticians are not currently part of the standard psychiatric healthcare team, yet lifestyle programs may be beneficial to improving cognitive and affective symptoms in individuals with mood disorders (Martin et al., 2016). This contrasts with the evidence that some specific nutrients display protective properties over mood fluctuations including vitamin C, magnesium and zinc. Specifically, low vitamin C or folate levels have been associated with increased mania and depressive symptoms, respectively (Martin et al., 2016; Martins et al., 2021). Other nutrients such as omega-3 polyunsaturated fatty acids, vitamin D, polyphenols and zinc demonstrate anti-inflammatory and neuroprotective actions, such as increasing neurotrophin levels in the brain (Godos et al., 2020; Martins et al., 2021). Additionally, the antioxidant actions of zinc, magnesium and B vitamins may counteract oxidative stress and mitochondrial dysfunction in mood disorders (Yang et al., 2020). Vitamin B12 has even been suggested as an effective adjunctive treatment for depression (Du et al., 2016). This is also true for some diets.

The Mediterranean diet has been repeatedly discussed as having a protective role on mood and cognition and is rich with the aforementioned vitamins and minerals through high consumption of fruits, vegetables, whole grains and fish. Adherence to the Mediterranean diet results in improved severity of depressive episodes and reduces the risk of MDD by about 30% (Adan et al., 2019; Martins et al., 2021; Marx et al., 2017; Perez, 2018). A potential mechanism for this diet is enhanced regulation of proinflammatory cytokines as well as increased gut microbiota diversity which directly correlates with depressive symptoms (Godos et al., 2020; Oh et al., 2020).

The role of the gut microbiome on mood is also important to consider in diet therapy. Emerging evidence indicates anti-inflammatory and anti-depressant properties of pre- and pro-biotic substances. Specifically, probiotics have been shown to improve depressive symptoms through downregulation of HPA activity and increased production of serotonin precursors (Martins et al., 2021). In addition, improved appetite has been demonstrated following probiotic supplementation in MDD patients (Kazemi, Noorbala, & Djafarian, 2020). Although more randomized clinical trials are needed, ‘Psychobiotics’ demonstrate potential as effective adjunctive treatments for EBPs of emotional eating or altered appetite (Kim & Shin, 2019; Noonan, Zaveri, Macaninch, & Martyn, 2020).

In addition to promotion of a healthy dietary lifestyle, it has been suggested in the literature that diet therapy should focus on distinguishing psychological cravings from physiological needs in order to gain control over food intake and limit maladaptive eating behaviors (Bernstein et al., 2015). Therefore, psychotherapy will be of benefit to emotional eating and food ‘addiction’ EBPs in mood disorders, as indicated in Table 2. Emotion-focused therapy is a psychotherapeutic technique which aims to gain control over emotional processes and may be beneficial to limiting emotional eating as a result of mood dysfunction. For example, emotion-focused therapy has been shown to increase affective control and reduce the incidence of maladaptive eating behaviors such as bingeing and purging in individuals with eating disorders (Osoro, Villalobos, & Tamayo, 2021). Similarly, cognitive behavioral therapy is already considered a standard treatment for mood disorders and has the potential to reduce psychological distress, improve personal coping mechanisms, and favor positive clinical outcomes regarding eating behavior (Murphy, Straebler, Cooper, & Fairburn, 2010).

**Table 2.** Main characteristics, biological substrates and potential treatment approaches for Eating Behavioral Phenotypes (EBPs) in mood disorders

EBP	(1) High appetite	(2) Low appetite	(3) Emotional eating
Prevalence	1/3 of individuals with depression BD depressive episodes Predominantly female	½ of depressed individuals BD hypomanic phases Melancholic depression Predominantly male	Prevalent in mood disorders with comorbid eating disorders (especially BED)
Characteristics	Interoceptive deficits Abnormal hunger perception Abnormal reward cues	Increased satiety Altered pleasure response Neurocognitive deficits	Overeating Disinhibition Binge eating
Biological Substrates	Altered insula and reward system activity Altered leptin and ghrelin levels Insulin resistance Altered immune function	Increased cortisol levels Altered immune function Altered neural activity in circuits governing mood and neurocognition	Psychological self-medication Altered serotonin activity (5-HTTLPR genotype)
Potential Treatments	GLP-1 agonists (Liraglutide, Semaglutide) Naltrexone and Bupropion Metformin GI lipase inhibitor (Orlistat)	Appetite stimulants (Dronabinol, Megestrol, Mirtazapine)	Serotonergic agents (SSRIs, TCAs, MAOIs) CBT Emotional-focused therapy Opioid antagonists
EBP	(4) Food ‘addiction’	(5) Unhealthy diet	(6) Circadian desynchronized
Prevalence	25% of depressed people Predominantly female	The majority of individuals with mood disorders Prevalent in SAD	The majority of individuals with mood disorders Comorbid NES
Characteristics	Increased impulsivity Increased food cravings Preference for highly palatable foods Altered inferred food pleasantness	Reduced protein intake Increased fat intake Increased intake of calorically dense foods Seeking and consuming sweets and refined carbohydrates Short-term enhancement of cognitive function	Irregular eating patterns Nocturnal eating Altered social activity (eating alone)
Biological substrates	Altered reward system activation Inflammation Abnormal neural activity in regions involved in food significance and reward valuation (OFC, insula) Increased C-reactive protein	Abnormal hormone signaling Altered serotonergic signaling	Neuroendocrine signaling Circadian clock genes
Potential treatments	Dopaminergic agents (Bupropione) Opioid antagonist (Naltrexone, Contrave) CBT Acamprosate	Dietetic counseling (Mediterranean, ketogenic, plant-based diets) Supplementation (B12, zinc, magnesium, polyphenols, omega-3, vitamins C and D)	CBT Rhythm therapy Time-restricted eating Light therapy

BD, Bipolar disorder; BED, Binge Eating Disorder; GLP-1, Glucagon-Like Peptide-1; GI, Gastrointestinal; SSRI, Selective Serotonin Reuptake Inhibitor; TCA, Tricyclic Antidepressant; MAOI, Monoamine Oxidase Inhibitor; CBT, Cognitive-Behavioral Therapy; SAD, Seasonal Affective Disorder; NES, Night Eating Syndrome; OFC, Orbitofrontal Cortex.

Finally, it is possible that interventions targeting circadian rhythms are beneficial to disordered mood and eating, including rhythm therapy and light therapy (Beauchamp & Lundgren, 2016). Bright light therapy involves the delivery of artificial light to the retina in order to gradually shift circadian patterns to the desired phase (Beauchamp & Lundgren, 2016). Similarly, rhythm therapy involves the stabilization of circadian patterns by modulating the timing of behaviors such as social activity and life events. Both of these methods have demonstrated efficacy in improving mood symptoms as well as potential for treating the circadian desynchronization of eating behavior (Haynes, Gengler, & Kelly, 2016; Oldham & Ciraulo, 2014).

Time-restricted eating has also demonstrated benefit for the circadian desynchronized EBP, defined as the strict separation of feeding and fasting windows in which food intake is limited to a 12–20-hour window during the active phase (Guerrero-Vargas et al., 2021; Longo & Panda, 2016). Time-restricted eating has already been explored for the treatment of circadian rhythm-related metabolic dysfunction (Longo & Panda, 2016; Manoogian & Panda, 2017). For example, one study showed that time-restricted eating was sufficient to prevent obesity even without affecting daily caloric intake (Oike et al., 2015). In addition, individuals being treated with lithium demonstrate greater regularity in eating and significant improvements in mood state when compared to patients not taking lithium (Buyukkurt et al., 2021).

Although emerging evidence suggests that time-restricted eating may improve eating rhythms and negative mood, more work is needed to identify the underlying mechanisms for this relationship. One study indicated that elevated adiponectin levels while fasting are associated with a reduced risk of adverse cardiovascular effects and may mediate the positive effects of calorie restriction or intermittent fasting for cardiometabolic health (Godos et al., 2020). In addition, fasting promotes the production of ketone bodies, which have been linked to improved energy metabolism and neuroprotection (Jensen, Wodschow, Nilsson, & Rungby, 2020). Time-restricted eating also promotes positive mood through the actions of neurotrophic factors, cytokines and neuroendocrine signaling (Igwe, Sone, Matveychuk, Baker, & Dursun, 2021). Despite these lines of evidence, more work is needed to confirm the underlying mechanisms responsible for improved mood symptoms following a time-restricted eating regimen.

With the vast heterogeneity of EBPs, it is important to note that time-restricted eating may not be appropriate for all individuals with mood disorders and maladaptive eating behaviors. For example, an individual with the circadian desynchronized EBP or high appetite EBP would benefit from this strategy while someone with a low appetite EBP and weight loss would not. Likewise, an individual with a comorbid ED such as anorexia nervosa or bulimia nervosa would also not be suitable due to the restrictive nature of this intervention. The need for different treatment approaches for each EBP reinforces the notion that phenotyping is beneficial to the identification of more effective and personalized treatment strategies for mood disorders. In addition, more work is needed to develop treatment protocols which address both mood and eating behavior.

From a transdiagnostic lens, multiple pharmacotherapies used in psychiatry may also prove useful in the treatment of maladaptive eating behavior in mood disorders, as outlined in Table 2. For example, appetite suppressants such as GLP-1 agonists (e.g. liraglutide), metformin, and orlistat may be implemented for the high appetite EBP (Chwastiak & Tek, 2014; Kanoski, Hayes, & Skibicka, 2016; Mansur et al., 2017; Park et al., 2018; Watson et al., 2019). In addition, appetite stimulants are already being adapted to psychiatric illnesses, including dronabinol, megestrol and mirtazapine to increase weight in cases of low appetite (Badowski & Yanful, 2018; Howard, Hossaini, Tolar, & Gaviola, 2019). Serotonergic agents may be useful to the emotional eating EBP as well as opioid antagonists which have demonstrated success in the treatment of binge eating (Glisenti, Strodl, King, & Greenberg, 2021; McElroy, Guerdjikova, Mori, & O'Melia, 2012). Other potential pharmacotherapies for EBPs include dopaminergic and opioid agents for the food ‘addiction’ EBP as well as nutritional supplementation for the unhealthy diet EBP (Martins et al., 2021; McElroy et al., 2012). When considering such psychopharmacological therapies, it is important to address the impact of medications on eating behavior. For example, second-generation antipsychotics are widely prescribed for BD but have been linked to poor appetite control, weight gain and the development of metabolic syndrome (Barton, Segger, Fischer, Obermeier, & Musil, 2020; Dayabandara et al., 2017). Considering a personalized medicine approach will be principal in moving forward with the development of treatment protocols for EBPs in mood disorders.

Although the characterized EBPs have yet to be empirically validated, the presence and burden of maladaptive eating behaviors in mood disorders is indisputable. Therefore, it is important to consider these proposed therapeutic strategies as important avenues for further investigation. More work is needed to confirm the therapeutic efficacy of these methods and to promote the personalization of pharmacological therapies for maladaptive eating behavior in mood disorders.

## Future directions and limitations

As eating behavior in mood disorders continues to be characterized and validated, EBPs will allow for enhanced development of alternative treatment approaches into standard clinical care. For example, specific diet therapies for the unhealthy diet EBP, ecological-momentary assessment techniques and time-restricted eating for the circadian desynchronized EBP, as well as psychotherapy for the food ‘addiction’ and emotional eating EBPs. The presence of EBPs in mood disorders has also been supported by specific pharmacotherapies and their role on reward aspects of BD treatment. One study indicated that individuals with BD taking olanzapine or quetiapine reported increased food cravings, specifically for sweet foods. In contrast, individuals taking valproate or lithium demonstrated fewer food cravings (Platzer et al., 2020). Despite this, mood stabilizers have also been repeatedly linked to weight gain (El-Khatib et al., 2007; Martin, Han, Anton, Greenway, & Smith, 2009). Thus, the characterization of EBPs may improve the precision with which pharmacotherapies are selected for individuals with mood disorders, based on knowledge of specific subtypes relevant to treatment response.

It is important to consider that extensive work is still needed in the characterization of EBPs in mood disorders and their applications to diagnostic criteria. It is possible that other eating behavior characterizations also exist in the mood disorder population. In fact, there is some evidence for a phenotype related to obsessive-compulsive eating behavior with changes in mood being correlated with orthorexic habits and obsessional or unhealthy fixations with eating (Lopes, Melo, & Dias Pereira, 2020). It is also important to note that the identified EBPs are not mutually exclusive. Some individuals with mood disorders may present with maladaptive eating behaviors that are overlapping between phenotypes, such as an individual with depression who simultaneously suffers from high appetite, emotional eating and an unhealthy diet.

The role of eating behavior in the pathophysiology of mood disorders cannot be fully elucidated until there is an increased understanding of the neurobiological relationship between feeding and mood. Specifically, the directionality of this relationship remains to be fully uncovered which may influence the nature of the aforementioned EBPs. For example, one study on the temporal relationship between disordered eating symptoms and depressive symptoms found that the onset of depressive symptoms predicted future bulimic episodes. The onset of bulimic episodes likewise predicted increased depressive symptoms (Presnell, Stice, Seidel, & Madeley, 2009). More recently, a large survey study found that, as depressive and anxiety symptoms increased, so did disordered eating behaviors in college students (Eck & Byrd-Bredbenner, 2021).

There is also importance in considering how the prominent overlap between mood disorders and EDs is relevant to the identified EBPs. It is known that EDs are more commonly diagnosed in individuals with mood disorders than in the general population and a comorbid diagnosis is associated with worse mood symptoms, poorer prognosis, and a higher burden of illness (Boulanger et al., 2018; Godart et al., 2015; Sander, Moessner, & Bauer, 2021; Woldeyohannes et al., 2016). Therefore, it is possible that EBPs may include cases in which a comorbid ED diagnosis is warranted. Changes in appetite are listed in the DSM-5 criteria for both EDs and mood disorders, although low appetite associated with depression is not usually as severe as that of anorexia nervosa (DeSocio, 2019). Conversely, maladaptive eating behaviors in the context of mood disorders may increase the risk of developing an ED later in the disease course. For example, it has been proposed that some individuals with EDs may express ED symptoms as a means to regulate mood instability (Henderson, Fox, Trayner, & Wittkowski, 2019). More work is needed to identify the mechanisms underlying the progression of EDs from maladaptive eating behavior in mood disorders, including how the simultaneous experiences of weight gain, body dissatisfaction, and potential compensatory behaviors play a role.

Some research has postulated the presence of a common etiology between mood disorders and EDs. although more evidence on the mechanistic underpinnings is needed (McAulay et al., 2019; Rossetti, Halfon, & Boutrel, 2014). It is possible that the reciprocal relationship between eating and mood may represent a main feature of the underlying pathophysiology of mood disorders, or alternatively, future research may indicate the existence of a third factor underpinning both mood and eating disorders (Presnell et al., 2009). For example, dysfunctional eating behavior cognitions, avoidant personality traits, perfectionism, insufficient tryptophan intake and polymorphisms of the serotonin transporter gene are linked to both EDs and mood disorders (6, 16).

It is evident that maladaptive eating behavior exists on a spectrum and requires more attention in the clinical assessment of mood disorders. Incorporating the characterization of EBPs into mood disorder care may help prevent the development of severe eating dysfunction, worsened mood symptoms and/or comorbid EDs. Considerable work is needed to develop treatment protocols which consider both disordered eating and mood simultaneously. In addition, future work should aim to empirically test these eating phenotypes and examine their associations with treatment outcomes and prognoses.

## Conclusion

Mood disorders are not simply illnesses of disordered mood. They are heterogeneous conditions, comprised of abnormalities in multiple overlapping systems, including metabolism, appetite and behavior. The classification of EBPs in the mood disorder population parallels the work of the Research Domain Criteria (RDOC) project which aims to conceptualize psychiatric diagnoses as a matrix of symptoms and their biological correlates opposed to symptom-based categories (Wildes & Marcus, 2015). This narrative review has described a variety of EBPs identified in the mood disorder literature to date, the proposed biological substrates involved and the implications for therapeutic strategies. Appetite-related EBPs manifest as two main subtypes and indicate dysfunctional energy regulation in mood disorders. In addition, the emotional eating EBP provides an explanation for the increased prevalence of obesity and binge eating in the mood disorder population in which patients may use food to offset negative affect. The food ‘addiction’ EBP is a reflection of how neural circuits regulating food intake and pleasure interact, manifesting as food craving and impulsivity. Meanwhile, the unhealthy diet EBP indicates how different diet compositions affect mood and the circadian desynchronized EBP reiterates the important relationship between internal clocks, metabolism and mental health. Overall, the classification of EBPs has the potential to increase etiological knowledge and the precision of diagnostic and treatment strategies tailored to each phenotype, contributing to increased quality of patient care.
